# Supplementary material for: Methyl Jasmonate- and Salicylic Acid-Induced Transcription Factor ZjWRKY18 Regulates Triterpenoid Accumulation and Salt Stress Tolerance in Jujube
Source: Int J Mol Sci. 2023 Feb 15;24(4):3899. doi: 10.3390/ijms24043899 (PMC9965381; doi:10.3390/ijms24043899)
Supplement: Supplementary file 1 [file ijms-24-03899-s001.zip › supplementary figures.pdf]

a

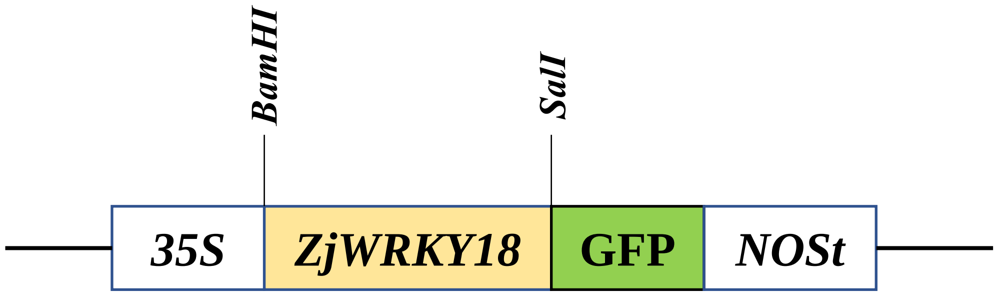

pCambia2300::*ZjWRKY18*-GFP

b

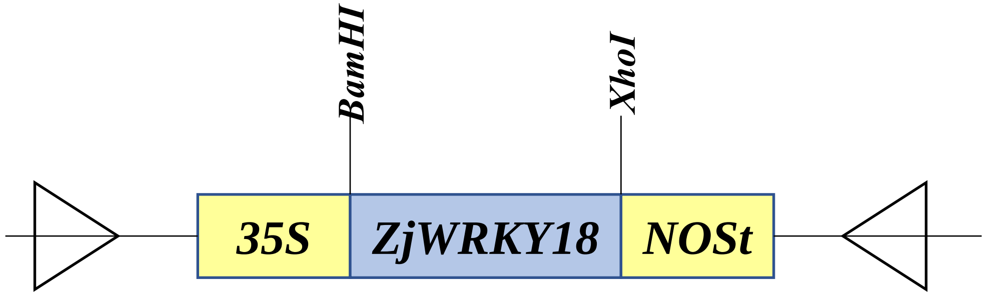

pTRV2::*ZjWRKY18*

Figure S1 pCambia2300-GFP and pTRV2 derived vector maps used for subcellular localization, overexpression (a) and VIGS (b) studies, respectively.

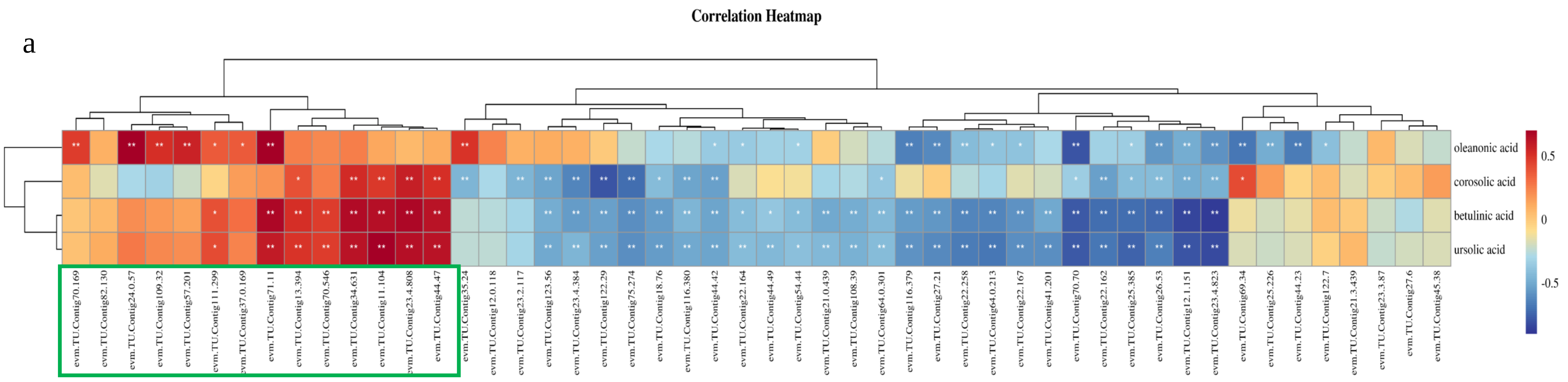

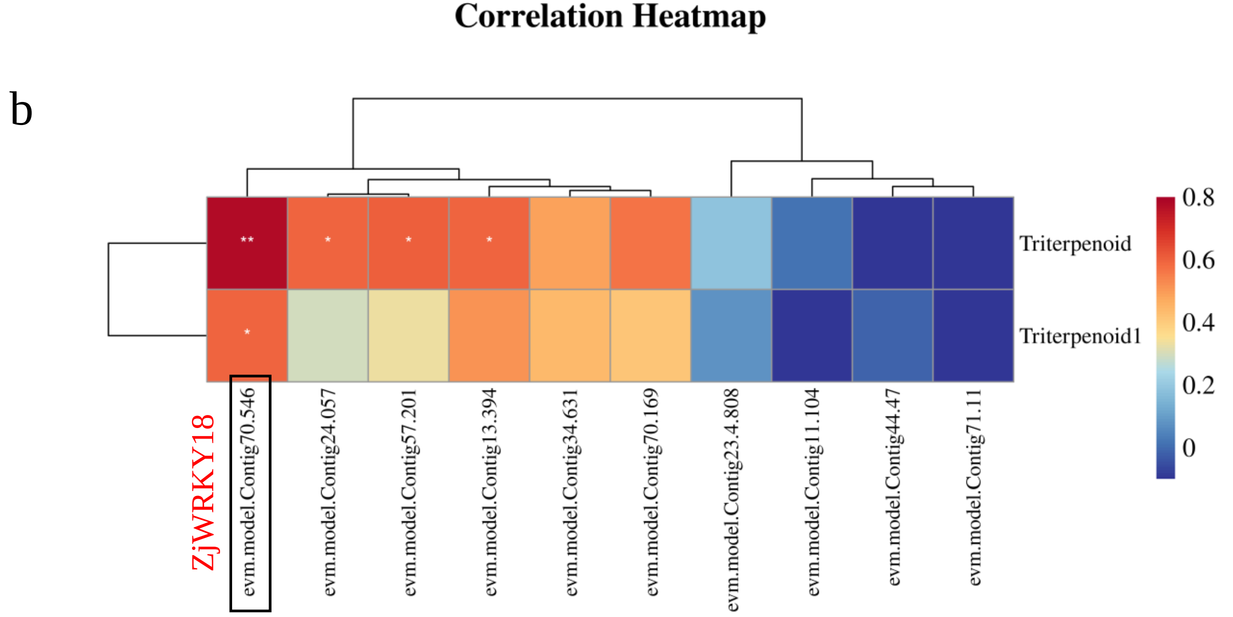

Figure S2. Correlation between WRKY gene expression and triterpene content. (a) Correlation analysis of WRKY gene expression screened by transcriptome and triterpene content; (b) Correlation between total triterpenoid content and gene expression after MeJA and SA treatments. Triterpenoid and triterpenoid1 represent two repetitions of the total triterpenoid content.

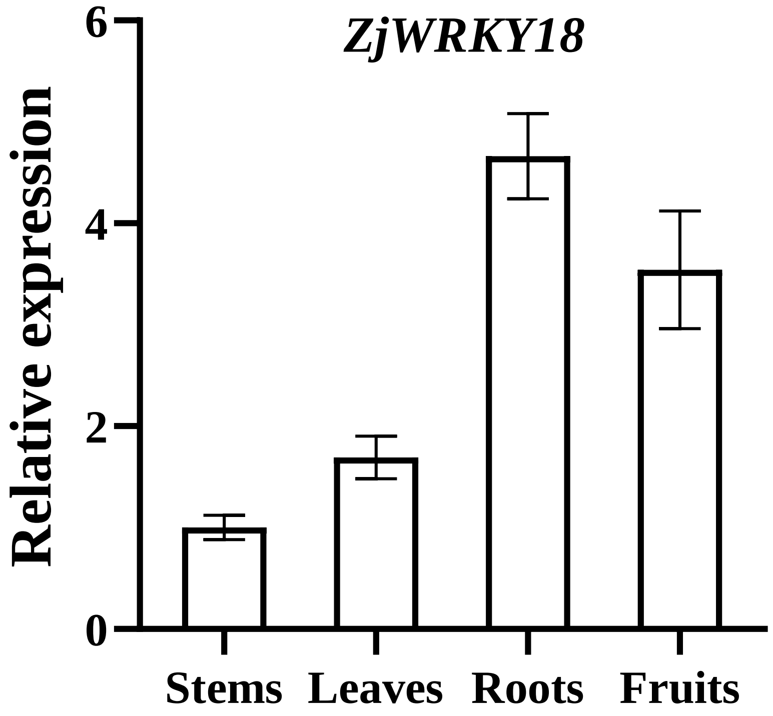

Figure S3. Relative expression of *ZjWRKY18* in different tissues of jujube seedlings and jujube fruits.

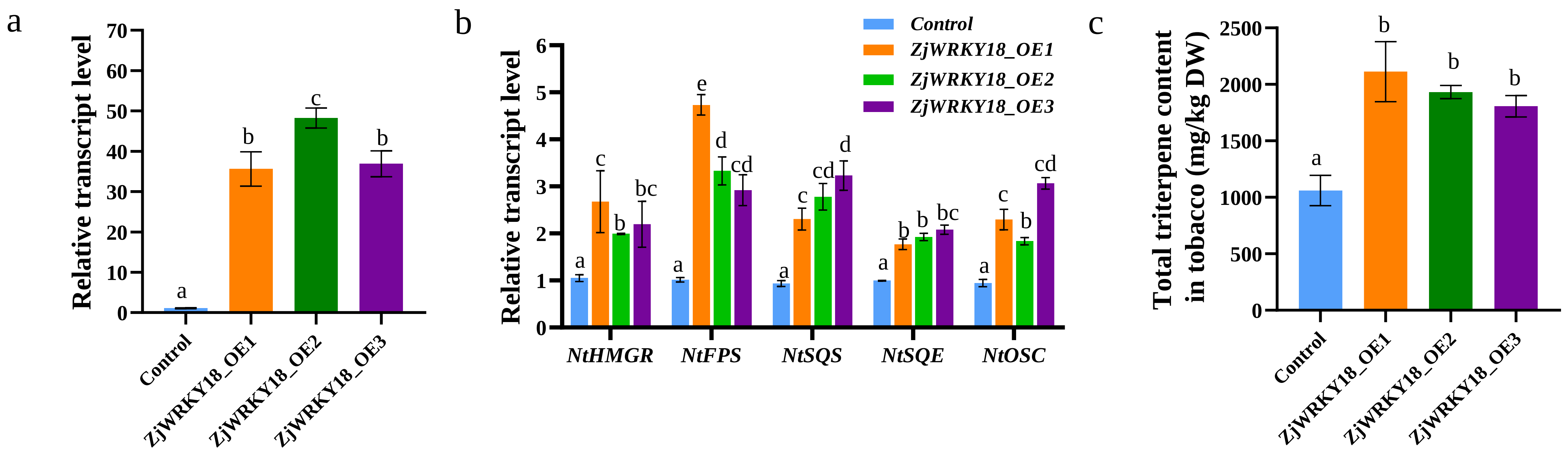

Figure S4. Effects of overexpression of *ZjWRKY18* on the expression level of triterpenoid synthesis pathway and total triterpenoids in tobacco. (a) Relative levels of ZjWRKY18 and mevalonate pathway genes in pC2300 control and ZjWRKY18-OE overexpression lines; (b) Expression of ZjWRKY18 and triterpenoid pathway genes in transgenic lines of tobacco; (c) Total triterpene content changes in OE-ZjWRKY18 leaves compared with control.

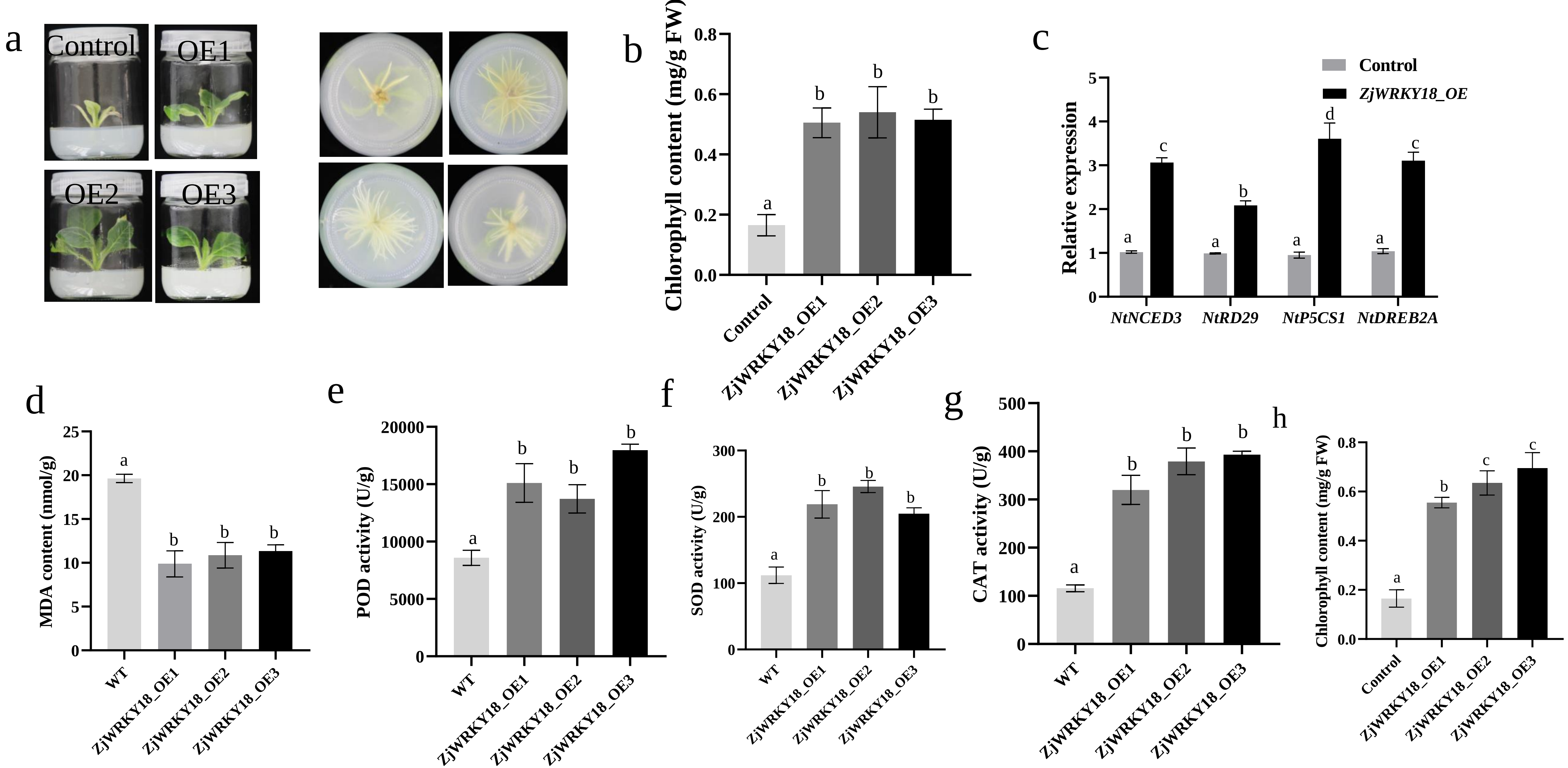

Figure S5. Effect of *ZjWRKY18* overexpression on defense-related genes and salt stress in tobacco. (a) Growth phenotypes of overexpressed ZjWRKY18 tobacco and control under salt stress; (b) The difference of chlorophyll content between overexpressed and control lines; (c) Expression levels of defense related-genes; (d-g) The analysis of MDA content and SOD, POD, and CAT activities in overexpressed and control lines.
